# Role of gut microbiome in suppression of cancers

**DOI:** 10.1080/19490976.2025.2495183

**Published:** 2025-04-20

**Authors:** Yao Xu, Zhaoyu Gao, Jiaying Liu, Qianqian Yang, Shunjiang Xu

**Affiliations:** a Central Laboratory, The First Hospital of Hebei Medical University, Shijiazhuang, P. R. China; b Department of Dermatology, Huashan Hospital, Fudan University, Shanghai, P. R. China; c The Key Laboratory of Neural and Vascular Biology, Ministry of Education, Shijiazhuang, P. R. China; d Hebei Key Laboratory of Brain Science and Psychiatric-Psychologic Disease, Shijiazhuang, P. R. China

**Keywords:** Gut microbiome, cancer, suppression

## Abstract

The pathogenesis of cancer is closely related to the disruption of homeostasis in the human body. The gut microbiome plays crucial roles in maintaining the homeostasis of its host throughout lifespan. In recent years, a large number of studies have shown that dysbiosis of the gut microbiome is involved in the entire process of cancer initiation, development, and prognosis by influencing the host immune system and metabolism. Some specific intestinal bacteria promote the occurrence and development of cancers under certain conditions. Conversely, some other specific intestinal bacteria suppress the oncogenesis and progression of cancers, including inhibiting the occurrence of cancers, delaying the progression of cancers and boosting the therapeutic effect on cancers. The promoting effects of the gut microbiome on cancers have been comprehensively discussed in the previous review. This article will review the latest advances in the roles and mechanisms of gut microbiome in cancer suppression, providing a new perspective for developing strategies of cancer prevention and treatment.

## 1. Introduction

The gut microbiome, often referred to as the “second genome,” comprises a diverse population of microorganisms, including bacteria, fungi, viruses, and archaea, that reside in the human gastrointestinal tract.^1,2^ The human gut is estimated to harbor over 100 trillion microbial cells, which possess a genetic repertoire, playing a vital role in human health and diseases.^3^ Several factors can influence the composition and function of the gut microbiota, such as dietary habits, host genetics and other host-related factors.^2,4^ Diet is one of the main drivers shaping the gut microbiota across the lifespan, with different dietary patterns and carbohydrate intakes having profound effects on gut microbial communities. For instance, diets rich in fats and low in fiber alter the microbial composition, reducing the abundance of short chain fatty acid (SCFA)-producing bacteria. In contrast, high-fiber diets promote the growth of beneficial microbes like *Akkermansia muciniphila*, which has been linked to improved intestinal health and anti-tumorigenic effects.^5^ Antibiotics can significantly alter the gut microbiota by reducing microbial diversity and disrupting the balance between beneficial and harmful microbes.^6^ Age is another factor, as the gut microbiota dynamically changes with aging, affecting microbial composition and diversity in host.^7,8^ Additionally, environmental conditions such as infections, medication, and hygiene, also have strong influences on the gut bacterial community.^9–11^ The gut microbes are not just passive inhabitants but actively participate in various physiological processes, including digestion, metabolic regulation, and immune system function. The gut microbiome is intricately linked with human health, influencing resistance to pathogens, immune system regulation, digestion, metabolism, epithelial cell proliferation, insulin resistance, and even behavioral and neurological functions.^12^ A balanced gut microbiome is crucial for maintaining the homeostasis of its host, while an imbalance, or dysbiosis of the gut microbiome, has been associated with various diseases or conditions, including inflammatory bowel disease, obesity, diabetes, and cancers.^13^

Multiple types of cancer, particularly malignant ones, pose significant health risks and are a leading cause of morbidity and mortality worldwide, with projections indicating nearly 10 million cancer-related deaths in 2022, and expected to rise to over 18 million by 2050.^14^ The incidence of new cancer cases is anticipated to increase from approximately 20 million in 2022 to nearly 35 million annually by 2050.^14^ The development of cancer is influenced by a combination of genetic factors and environmental exposures, as well as the disruption of homeostasis, leading to the transformation of normal cells into cancerous cells over time.^15^ Homeostasis is a fundamental concept in physiology that refers to the self-regulating processes by which an organism maintains a stable internal environment while adjusting to changing external conditions.^16^ It is a dynamic process that can change internal conditions as required to survive external challenges. Gut microbiome interacts with immune and metabolic systems, playing a significant role in maintaining human homeostasis through various metabolic, immune, and protective functions.^17,18^ The relationship between the gut microbiome and human homeostasis is a complex and dynamic interaction that plays a crucial role in maintaining health and has a significant impact on the development of cancers.

Recent advances in sequencing technologies and system biology approaches have provided unprecedented insights into the complex interactions between gut microbes and cancer. The gut microbiome plays a dual role in cancer that it can act as both a tumor-promoting mechanism and a tumor-suppressive mechanism, influencing cancer initiation and progression. A growing body of evidence suggests that microbial dysbiosis contributes to tumorigenesis through chronic inflammation, altered metabolism, and immune modulation.^19^ Conversely, certain microbial populations and their metabolites can enhance the suppression of cancers, supporting the development of novel microbiota-based therapeutic strategies.^20^ The roles of gut microbiome in cancer suppression include inhibiting the occurrence of cancers, delaying the progression of cancers and boosting the therapeutic effect on cancers. This review aims to provide a comprehensive analysis of the roles and mechanisms by which the gut microbiome influences cancer initiation and development, with a focus on its role in suppressing cancer and its implications for cancer prevention, therapeutics, and personalized medicine.

## 2. Alterations of gut microbiome composition in cancers

Numerous clinical studies and animal experiments have shown that the composition of the gut microbiome changes during the occurrence and development of tumors, leading to a dysbiosis in the gut microbes.^21,22^ The dysbiosis of gut microbiota, defined as an imbalance in the gut microbial community, is a hallmark of many diseases, including various cancers (Table 1). In healthy individuals, gut microbiota exhibit a balanced composition dominated by beneficial microbes such as *Lactobacillus*, *Bifidobacterium*, and *Faecalibacterium prausnitzii*.^55,56^ These beneficial microbes are critical for maintaining gut barrier integrity, regulating immune responses, and producing SCFAs with anti-inflammatory properties.^57,58^ However, in patients with cancer, the gut microbiota dysbiosis is characterized by decreased diversity and overgrowth of pathogens. As shown in Table 1, loss of microbial diversity is a common feature in patients with colorectal cancer (CRC), breast cancer, and pancreatic cancer. For instance, CRC patients show a significant reduction in *Bifidobacterium* and an enrichment of *Fusobacterium nucleatum*, a bacterium associated with pro-inflammatory and pro-tumorigenic activity.^59^ In addition, pathogenic species such as *Escherichia coli* and *Clostridium septicum* produce genotoxins that induce DNA damage, promoting tumor initiation.^60^ The dysbiotic microbiota creates a microenvironment conducive to tumor growth by disrupting mucosal barriers, modulating immune responses, and influencing metabolic pathways. On the other hand, some other gut bacteria are protective against the occurrence and progression of cancers. Examples include *Clostridium butyricum*, *Streptococcus thermophilus*, and *Lacticaseibacillus paracasei*.^58,61–63^ These bacteria produce beneficial metabolites such as butyrate, which can impede tumor development and progression.^64,65^ Recently, a lot of reviews have deeply and extensively discussed how the gut microbiome promotes the occurrence and progression of cancers.^29,35,66^ Therefore, this review will focus on discussing the roles of gut microbiome in enhancing the suppression of cancers and its underlying mechanisms.

**Table 1.** Alterations of gut microbiome composition in cancers.

Type of Cancer	Sample size	Detection method	Detection objects	Organism	Effect	Expression pattern	Mechanism Pathways	Reference
Colorectal cancer	*In vitro* study			*Enterotoxigenic Bacteroides fragilis (ETBF)*	Promote	Enriched	ETBF/miR-149-3p pathway	Cao et al. 2021^23^
	30 CRC patients and 30 healthy control	Metagenomic sequencing	Fecal samples	*Fusobacterium nucleatum*			Activating a TLR4/Keap1/NRF2 signaling axis	Kong et al. 2021^24^
	*In vitro* study						Release of ADP-Heptose and ALPK1/TIFA Axis Activation	Martin-Gallausiaux et al. 2024^25^
	*In vitro* study						Inducing Epithelial-Mesenchymal Transition (EMT)	Casasanta et al. 2020^26^
	*In vitro* study			*Lactobacillus johnsonii*	Inhibit	Decreased	Modulating the inflammasome and kynurenine pathway	Teixeira et al. 2018^27^
	*In vitro* study						Enhancing the integrity of the gut barrier by interacting with tight junction proteins such as JAM-4	Bai et al. 2022^28^
	60 male BALB/c	16S rDNA sequencing	Mice fecal pellets	*Parabacteroides goldsteinii*			Changing the gut microbiota composition	Yuan et al. 2022^29^
Colorectal cancer	*In vitro* study			*Butyrate-producing bacteria*	Inhibit	Decreased	Inhibiting mTOR/S6K1 Signaling Pathway	Cao et al. 2019^30^
	*In vitro* study			*Butyrate-producing bacteria*			Activating GPR109a-AKT Signaling Pathway	Geng et al. 2021^31^
	30 female four-week-old Apcmin/ + mice	16S rRNA gene sequencing	Mice fecal pellets	*Clostridium butyricum*			Suppressing the Wnt/β-catenin signaling pathway	Chen et al. 2020^32^
	22 CRC patients	16S rRNA sequencing	Fecal samples	*Akkermansia muciniphila*			Inducing TLR2/NLRP3 pathway	Wang et al. 2020^33^
	42 CRC patients	qRT-PCR	Fecal samples	*Akkermansia muciniphila*			Inhibiting tryptophan-mediated AhR/β-catenin signaling	Zhang et al. 2023^34^
	*In vitro* study			*Akkermansia muciniphila*			Protein Post-Translational Modification (PTM)	Jiang et al. 2023^35^
	*In vitro* study			*Akkermansia muciniphila*			Inducing apoptosis via The TRAIL-mediated apoptosis pathway	Meng et al. 2020^36^
	*In vitro* study			*Lactobacillus intestinalis*			The Activation of NOD1/NF-κB signaling pathway	Sun et al. 2025^37^
Gastric Cancer	39 GC patients	16s rRNA gene sequencing	Fecal samples	*Streptococcus anginosus*	Promote	Enriched	Direct interactions with gastric epithelial cells in TMPC-ANXA2-MAPK axis	Fu et al. 2024^38^
Gastric Cancer	*In vitro* study	16s rRNA gene sequencing	Fecal samples	*Helicobacter pylori (H. pylori)*	Promote	Enriched	The activation of JAK-STAT signaling	Li et al. 2022^39^
Liver Cancer	HCC mouse models	16s rRNA gene sequencing	Mice fecal pellets	*Bacteroides acidifaciens, Odoribacter laneus, Odoribacter splanchnicus*	Inhibit	Decreased	Upregulated interferon-γ expression of NK and T cells via the gastrointestinal microbiota -AMPK-mTOR axis	Pan et al. 2023^40^
	20 healthy controls and 26 patients	16s rRNA gene sequencing	Fecal samples	*Bacteroides, Faecalibacterium Roseburia, Subdoligranulum*	Inhibit	Decreased	Inhibit ferroptosis by regulating the ALK5/NOX1 axis	Zhang et al. 2024^41^
Esophageal Cancer	30 ESCC patients/4NQO-inducible mouse	16s rRNA gene sequencing	Fecal samples	*Bacteroides*	Promote	Enriched	TLR4/Myd88/NF-κB pathway activation	Wu et al. 2024^42^
Breast Cancer	42 breast cancer patients and 40 controls	16S rRNA sequencing	Fecal samples	*Prevotella copri*	Promote	Enriched	Inactivation of AMPK via UHRF1-mediated negative regulation	Su et al. 2024^43^
Ovarian Cancer	20 OC patients, 20 patients with EBOT, and 20 healthy controls	16S rRNA sequencing	Fecal samples	*Actinobacteria, Bifidobacterium, Ruminococcaceae_Ruminococcus, and Collinsella*	Inhibit	Decreased	Regulating Hedgehog signaling pathway	Hu et al. 2023^44^
	40 patients with OC and 40 patients	16S rRNA sequencing	Fecal samples	*Akkermansia*	Inhibit	Decreased	CD8^+^ T cell activation	Wang et al. 2022^45^
Prostate cancer	35 prostate cancer patients	16S rRNA sequencing	Fecal samples	*Proteobacteria*	Promote	Enriched	Activating NF-κB-IL6-STAT3 axis	Zhong W et al. 2022^46^
	Prostate cancer mice	16S rRNA sequencing	Mice fecal pellets	*SCFA-producing bacteria*	Promote	Enriched	Via IGF1 Signaling	Matsushita M et al. 2021^47^
Lung Cancer	29 NSCLC patients	16S rRNA sequencing	Fecal samples	*Actinomycetota、 Euryarchaeota*	Promote	Enriched	DNA synthesis and Translesion	Dora et al. 2024^48^
	In vitro study			*Paenibacillus odorifer*	Inhibit	Decreased	Releasing protective factor acetate	Chen et al. 2024^49^
Melanoma	20 Melanoma and 16 healthy controls	16S rRNA sequencing	Fecal samples	*Clostridiales*	Promote	Enriched	Inducing tumoral invasion by the activation of the EMT signaling pathway	Vitali F et al. 2022^50^
Pancreatic cancer	GF-Rag1 − / − mice	qPCR	Mice fecal pellets	*gut microbiota*	Promote	Enriched	Inhibiting intratumoral NK cell infiltration and activation	Yu et al. 2022^51^
	63 Pancreatic ductal adenocarc inoma patient	16S rRNA sequencing	Fecal samples	*Lactobacillus spp.*	Inhibit	Decreased	NK cell-mediated immunity	Liang et al. 2025^52^
Lymphoma	30 patients and 20 healthy controls	Shotgun metagenomic sequencing	Fecal samples	*Faecalibacterium prausnitzii*	Inhibit	Decreased	Dampening the JAK-STAT pathway	Shi et al. 2024^53^
	77 patients and 41 healthy controls	Shotgun metagenomic sequencing	Fecal samples	*Eubacterium rectale*			Alleviating the TNF-induced TLR4/MyD88/NF-κB axis	Lu et al. 2022^54^

## 3. Gut microbiome inhibits the initiation of cancer

### 3.1. Modulation of immune homeostasis

**Figure 1.**
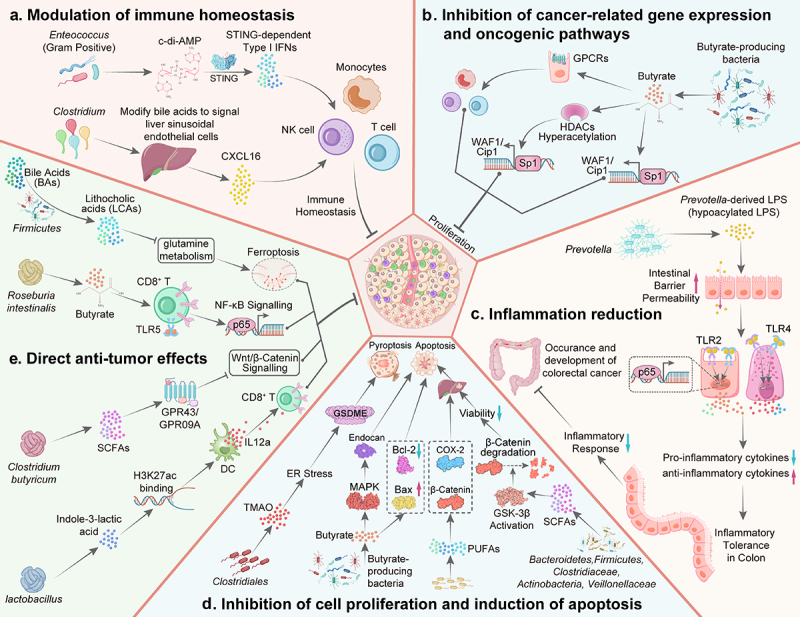
The mechanisms of gut microbiome inhibiting the initiation of cancers. A. Modulation of immune homeostasis: (1) Microbiota-derived cyclic di-AMP activates STING-dependent type I IFN production, leading to activation of innate immunity (including monocytes, NK cells and DCs); (2) *Clostridium* species modified bile acids to signal liver sinusoidal endothelial cells to produce CXCL16, a chemokine that recruits NKT immune cells to perform antitumor surveillance in the liver. B. Inhibition of cancer related gene expression and oncogenic pathways: (1) Butyrate inhibits HDACs, inducing histone hyperacetylation to activate p21/WAF1 and drive G1 arrest; (2) Butyrate activates WAF1 via Sp1 sites (p53-independently), halting cell cycle and tumor proliferation; (3) SCFAs activate GPCRs, such as FFAR3/2 and GPR109A, in gut/immune cells, leading to beneficial effects on cancer prevention. C. Inflammation reduction: Hypoacylated LPS derived from intestinal *Prevotella* exhibits weak TLR4 excitability and induces inflammatory tolerance in intestinal epithelial cells, thereby inhibiting the onset of CRC. D. Inhibition of cell proliferation and induction of apoptosis: (1) *Clostridiales* produced TMAO induced GSDME-mediated pyroptosis in tumor cells by activating the endoplasmic reticulum stress kinase PERK and thus enhanced CD8^+^ T cell-mediated antitumor immunity in TNBC. (2) Butyrate induces apoptosis (Bax and Bcl-2) and activates MAPK signaling to suppress proliferation/migration via endocan regulation, inhibiting cancer progression; (3) Certain SCFAs, such as PUFAs, inhibit hepatocellular carcinoma (HCC) growth by blocking β-catenin and cyclooxygenase-2 (COX-2); (4) SCFAs reduce cell viability and induce apoptosis in HCC cell lines through activation of GSK-3β, leading to β-catenin degradation. (E) Direct anti-tumor effects: (1) *Firmicutes* convert bile acids to lithocholic acid (LCA), which suppresses gallbladder cancer (GBC) by targeting GLS-glutamine metabolism to trigger ferroptosis; (2) Butyrate (*Roseburia intestinalis*) binds TLR5 on CD8^+^ T cells, activating NF-κB to boost activity; (3) *Lactobacillus plantarum* L168 and indole-3-lactic acid enhance IL12a production in dendritic cells via H3K27ac enrichment at IL12a enhancers, priming CD8^+^ T cell immunity to suppress tumor growth. (4) *Clostridium butyricum* inhibits the Wnt/β-catenin pathway, reshapes gut microbiota, elevates SCFAs, and activates GPR43/109A to suppress colorectal tumorigenesis.

Disruption of host homeostasis, including immune homeostasis, facilitates tumorigenesis in various tissues and organs. Gut microbiome plays a crucial role in maintaining immune homeostasis by influencing the balance between pro-inflammatory and anti-inflammatory responses.^67^ This balance is essential for preventing excessive inflammation, which can lead to cancer development.^68^ Cancer immunosurveillance refers to the innate and adaptive immune system’s ability to detect and eliminate recently transformed malignant cells before they can develop into clinically detectable tumors.^69^ This process begins with the recognition and destruction of tumor cells by innate immune cells, such as natural killer (NK) cells and dendritic cells (DCs).^67^ Microbiota-derived cyclic di-AMP activates STING-dependent type I IFN production, leading to activation of innate immunity (including monocytes, NK cells and DCs).^70^ The gut microbiome influences the recruitment and activation of immune cells such as CD4^+^ T cells and dendritic cells, which are essential for the immune surveillance and elimination of cancer cells.^71,72^ Certain bacteria, such as *Clostridium* species, modify bile acids to signal the liver sinusoidal endothelial cells to produce CXCL16, a chemokine that recruits natural killer T (NKT) cells to perform antitumor surveillance in the liver.^73^ These immune cells are critical in the antitumor surveillance of multiple organs, as they can directly target and eliminate tumor cells. It is implicated that gut microbiome suppress the initiation of cancer via modulating immune homeostasis to enhance the function of immune surveillance (Figure 1(a)).

### 3.2. Inhibition of cancer related gene expression and oncogenic pathways

The occurrence of cancer involves the abnormal expression of multiple oncogenes and other cancer-related genes. Some metabolites of gut microbiota, such as SCFAs, inhibit the expression of genes involved in DNA replication and cell cycle/proliferation in human CRC cells, exerting antiproliferative activity via different pathways.^74^ Among them, butyrate is known to inhibit histone deacetylases (HDACs), leading to histone hyperacetylation. This hyperacetylation facilitates the transcription of genes involved in cell cycle regulation and apoptosis, such as p21/Cip1 and WAF1/Cip1, which are crucial for cell cycle arrest at the G1 phase.^75,76^ Beyond HDAC inhibition, butyrate also affects other epigenetic modifications and gene expression profiles, contributing to its overall antitumorigenic effects.^77^ This includes the activation of specific gene promoters like WAF1/Cip1 through Sp1 sites, independent of p53 status.^78^ The induction of these proteins helps in halting the cell cycle, thereby inhibiting tumor cell proliferation.^79^ SCFAs can also activate G protein-coupled receptors such as FFAR3, FFAR2, and GPR109A.^80–82^ These receptors are expressed in the gut epithelium and immune cells and play roles in modulating gut health and immune responses. Activation of these receptors by SCFAs can lead to beneficial effects on gastric health and cancer prevention.^83^ Therefore, some metabolites of gut microbiome can exert its anti-tumor roles through inhibition of cancer-related gene expression (Figure 1(b)).

### 3.3. Inflammation reduction

Gut dysbiosis is often associated with increased inflammation, which is a known risk factor for cancer development.^44,84^ The onset of multiple cancers, such as CRC and breast cancer, depends on local or systemic long-term chronic inflammatory responses. The intestinal metabolite tyrosol exhibits antitumor effects by inhibiting HIF-1α/NF-κB signaling pathway activation, leading to a reduction in the levels of ROS and inflammatory factors.^85^ Gut microbiome modulates a host’s inflammation by production of several substances, including lipopolysaccharides (LPS) and SCFAs. LPS is the main component of Gram negative bacteria, also known as endotoxins, which can results in severe inflammation and immune responses. The number of lipid A acyl chains is an important determinant of immune activation. Therefore, LPS derived from gut bacteria has a double-edged sword effect. Highly acylated LPS (such as *E. coli* LPS) is known as pro-inflammatory LPS. Low acylated LPS (such as *Bacteroidetes* LPS), known as anti-inflammatory LPS, exhibits weak TLR4 excitability and induces immune and inflammatory tolerance.^86^ Our recent research confirms that LPS derived from intestinal *Prevotella* is a low acylated LPS that can induce inflammatory tolerance in intestinal epithelial cells, thereby inhibiting the onset of CRC in the presence of Alzheimer’s disease (AD).^87^ The composition of gut microbiota is changing dynamically with aging.^88^ Some specific genera of gut microbiota is enriched, such as *Prevotella*, it can simultaneously promote AD onset and inhibit CRC tumorigenesis. The key mechanism involves a dual role of the gut microbiota – derived LPS in the brain and the intestine.^87^ These results indicate that inflammation can be reduced by restoring a balanced gut microbiome, thereby decreasing the risk of cancer occurrence (Figure 1(c)).

### 3.4. Inhibition of cell proliferation and induction of apoptosis

Some gut microbiome metabolites exert its anti-cancer roles via activation of apoptotic pathways. For instance, *Clostridiales* produced TMAO induced GSDME-mediated pyroptosis in tumor cells by activating the endoplasmic reticulum stress kinase PERK and thus enhanced CD8^+^ T cell-mediated antitumor immunity in TNBC in vivo.^89^ Intestinal bacteria-derived butyrate induces apoptosis in cancer cells by modulating various signaling pathways and protein expressions.^30^ It has been shown to increase the expression of pro-apoptotic proteins like Bax and decrease the expression of anti-apoptotic proteins like Bcl-2.^90^ Additionally, butyrate can activate the MAPK signaling pathway, which further suppresses proliferation and migration by regulating endocan expression.^91^ Similar to butyrate, certain SCFAs can inhibit cell proliferation and induce apoptosis in cancer cells, such as omega-3 polyunsaturated fatty acids (PUFAs), and have been shown to inhibit hepatocellular carcinoma (HCC) growth by blocking β-catenin and cyclooxygenase-2 (COX-2).^92,93^ These SCFAs reduce cell viability and induce apoptosis in HCC cell lines through mechanisms involving the activation of GSK-3β leading to β-catenin degradation^94^ (Figure 1(d)). Although the specific mechanisms may differ between SCFAs, their ability to suppress cell growth/proliferation and induce apoptosis is crucial for preventing cancer development.

### 3.5. Direct anti-tumor effects

Certain gut bacteria produce anti-carcinogenic compounds with direct anticancer properties. For instance, lithocholic acid, butyrate, and cadaverine have been identified as metabolites that can inhibit tumor cell growth directly.^95–97^ In addition, *lactobacillus* has been shown to inhibit the proliferation of cancer cells in vitro and reduce tumor formation rates in animal models through the modulation of gut microbiota and metabolites.^98^ Similarly, *Clostridium butyricum* produces butyrate, which inhibits intestinal tumor development by modulating Wnt signaling.^32^ Some other SCFAs, including acetic acid and propionic acid, influence the composition and function of the gut microbiome (Figure 1(e)). This modulation can lead to a reduction in carcinogenic factors and an increase in beneficial bacteria that produce anti-cancer compounds.^99^ This indicates that the complex interactions between gut bacteria are crucial for maintaining gut microbiome balance and preventing the occurrence of cancers.

## 4. Gut microbiome delays cancer progression

### 4.1. Influence on the tumor microenvironment

**Figure 2.**
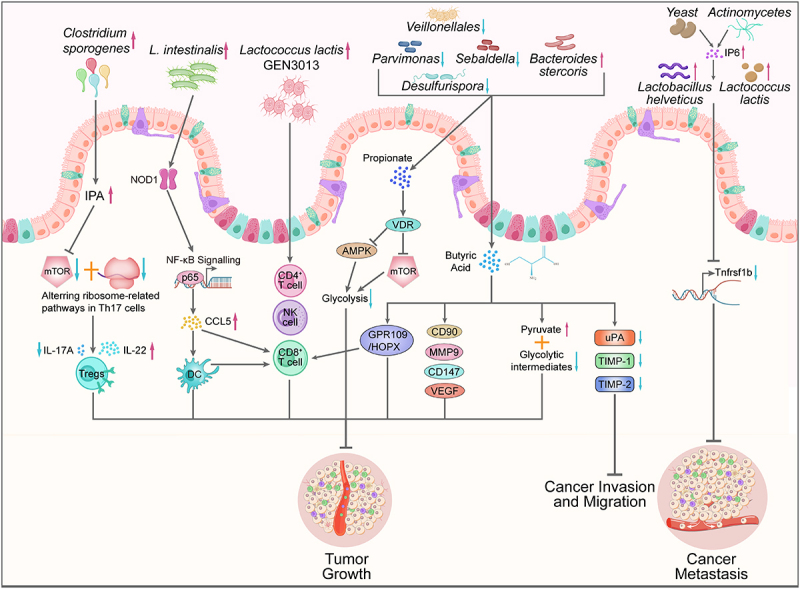
The mechanisms of gut microbiome delaying cancer progression. *Clostridium sporogenes*-derived IPA suppresses IL-17A in Th17 cells via mTOR/ribosome pathways, while its BCFAs/SCFAs enhance Treg activity and IL-22 production. *L. intestinalis* effectively suppressed tumor growth in CRC mice through increasing the infiltration of immune cells in the TME, particularly DCs, through NOD1/NF-κB signaling pathway. *Lactococcus lactis* GEN3013 boosts cytotoxic immune cells (CD4^+^/CD8^+^ T, NK) in TME; Propionates derived from several gut bacteria activate the AMPK/mTOR pathway through the vitamin D receptor (VDR), inhibit glycolysis in CRC cells. Butyrate exerts its inhibitory effects on tumor growth by enhancing CD8^+^ T cell cytotoxicity through GPR109A/HOPX signaling, inducing lung cancer cell cycle arrest (cell cycle proteins/CD90) and apoptosis, modulating thermal apoptosis/metastasis proteins (CD147/VEGF/MMP-9), and reprogramming cancer metabolism via pyruvate accumulation and glycolysis suppression, Butyrate can also curb the tumor cell invasion/migration by modulating proteolysis via suppressing uPA and enhancing TIMP-1/2 activity. Finally, IP6 from yeast/actinomycetes inhibits the expression of liver metastasis-linked Tnfrsf1b by restoring the abundance of *L. helveticus/L. lactis*.

The gut microbiome influences the tumor microenvironment (TME), which is crucial for cancer progression (Figure 2). Alterations in the gut microbiome due to dietary interventions can lead to widespread changes in the tumor immune microenvironment.^100^ For instance, mice fed a high-fiber diet showed enhanced antitumor immune responses characterized by increased fractions of B lineage cells, cDC1s, and M1-like macrophages, and reduced levels of activated cDC2s, M2 macrophages, and polymorphonuclear myeloid-derived suppressor cells.^101^ The gut microbiome influences the immune response in gastric cancer through triggering T-cell responses to bacterial antigens that can cross-react with tumor antigens, engaging pattern recognition receptors that mediate pro-immune or anti-inflammatory effects, and producing small metabolites that have systemic effects on the host.^83^ Interestingly, these differential effects of gut microbiome are highly strain-specific. For example, *Lactococcus lactis* GEN3013 inhibits tumor growth by regulating tumor angiogenesis and directly inducing cancer cell death, and *Bifidobacterium spp*. improve the function of dendritic cells (DCs), which are crucial for activating T cell responses against tumors.^102^ Enhanced DC function leads to better CD8^+^ T cell priming and accumulation in the TME, which is essential for effective immune surveillance.^103^ A recent study reported that *Lactobacillus intestinalis* (*L. intestinalis*) effectively suppressed tumor growth in CRC mice through increasing the infiltration of immune cells, particularly DCs, in the TME, which involved in the NOD1/NF-κB signaling pathway.^37^ Gut microbiome can alter nutrient availability and metabolic activity within the TME. For instance, specific microbial metabolites can influence the availability of SCFAs and secondary bile acids, which have been linked to CRC risk and progression.^104^ SCFAs can also regulate the number and function of regulatory T cells (Tregs) in the gut, which are critical for regulating intestinal inflammation and remodeling tumor immune microenvironment to delay cancer progression.^105^ This indicates that targeting the gut microbiome to regulate the TME will be an effective strategy for delaying cancer progression.

### 4.2. Metabolic reprogramming

Some metabolites of the gut microbiome, such as butyrate and propionates, influence the metabolic profile of cancer cells by promoting the accumulation of pyruvate and reducing glycolytic intermediates^67^ (Figure 2). Gut microbiome produced propionates activate the AMPK/mTOR pathway through the vitamin D receptor (VDR), inhibit glycolysis in CRC cells, and promote immunosuppressive function of regulatory T cells (Treg).^106^ This metabolic remodeling is associated with the inhibition of the Warburg effect, a characteristic metabolic shift in cancer cells toward increased glucose consumption and lactate production.^107^ By favoring energetic metabolism over anaerobic glycolysis, butyrate helps in suppressing tumor cell proliferation and progression.^108^ These researches suggest that SCFAs produced by gut microbiome may inhibit cancer progression through metabolic reprogramming.

### 4.3. Regulation of apoptosis and pyroptosis

Modulation of cell death, such as apoptosis and pyroptosis, serves as a fundamental approach in cancer therapy. SCFAs produced by gut bacteria, particularly butyrate, have been shown to suppress tumor growth and metastasis by regulating the cell cycle and inducing apoptosis in cancer cells.^109^ This is achieved by inducing cell cycle arrest and early apoptosis in lung cancer cells such as A549, as demonstrated by sodium butyrate (SB) treatment.^110^ Additionally, SCFAs can also inhibit proliferation, migration, and invasion of lung cancer cells by inducing apoptosis and G1/S arrest.^111^ This result suggests a potential mechanism through which SCFAs could inhibit cancer progression. Although not directly linked to gastric cancer in the provided evidence, the regulation of pyroptosis, a form of programmed cell death, has been suggested as another potential mechanism by which gut microbiota might influence cancer progression. The restoration of gut microbiota has been shown to inhibit gastric cancer progression by regulating pyroptosis.^112,113^ Wang et al. have reported that the microbial metabolite trimethylamine N-oxide (TMAO) enhances CD8^+^ T cell-mediated antitumor immunity in triple-negative breast cancer (TNBC) by inducing pyroptosis in tumor cells.^89^ This suggests that the gut microbiota could influence cancer cell survival and death mechanisms, potentially contributing to the inhibition of tumor growth and metastasis (Figure 2).

### 4.4. Inhibition of cancer invasion and metastasis

Tumor invasion and metastasis are hallmarks of cancer progression, involving the complex interplay between cancer cells and their surrounding microenvironment.^114^ Proteolytic enzymes, such as matrix metalloproteinases (MMPs) and urokinase plasminogen activator (uPA), play critical roles in tumor invasion and metastasis by degrading the extracellular matrix (ECM) and facilitating cancer cell migration.^115^ Butyrate regulates proteolytic activities by inhibiting uPA and stimulating the activity of tissue inhibitor matrix metalloproteinases (TIMP)-1 and TIMP-2 (Figure 2). This regulation reduces cell invasion and migration, both of which are critical steps in tumor progression.^116^ Gut bacteria produced SCFAs, particularly butyrate, have been shown to suppress specific genes involved in tumor growth and metastasis by modulating the cell cycle in cancer cells.^109^ For instance, sodium butyrate (SB) treatment has been demonstrated to induce cell cycle arrest and early apoptosis in lung cancer cells, such as A549 cells.^110^ Moreover, SCFAs can inhibit the proliferation, migration, and invasion of lung cancer cells by inducing apoptosis and causing G1/S cell cycle arrest.^111^ These findings highlight a potential mechanism by which SCFAs can inhibit cancer progression. Additionally, certain compounds like inositol hexaphosphate (IP6), a natural compound composed of inositol and six phosphate ions and rich in the form of phytic acid calcium magnesium salt in *yeast* and *actinomycetes*, have been shown to reduce CRC metastasis by mediating interactions between gut microbiota and host genes.^117^ This interaction influences gene expression related to metastasis, such as the Tnfrsf1b gene, which is negatively correlated with the abundance of *Lactobacillus helveticus* in the gut of host.^118^ This indicates that the gut microbiome and its metabolites play an important role in inhibiting tumor invasion and metastasis (Figure 2), which is of great significance for improving cancer treatment outcomes.

## 5. Gut microbiome boosts the therapeutic effect of cancer (Table 2)

### 5.1. Enhancement of immune checkpoint blockade effects

**Table 2.** Clinical trials investigating gut microbiome modulation to improve cancer therapy outcomes.

Cancer types	Sample size	Detection method	Biomarker/Treatment	Microbial interventions	Primary Outcome Measures	Reference
Colorectal cancer	CT26 syngeneic tumor modeling in BALB/c mice (*n* = 30)	16S rDNA sequencing	Biomarker	*Oscillibacter*	Reduce intestinal inflammation by regulating immune cells	Jia et al. 2024 ^119^
Non-small cell lung cancer	NSCLC treated with first- or second-line ICIs (*n* = 338)	Shotgun-metagenomics-based microbiome profiling	Treatment (NCT04567446)	*Akkermansia muciniphila*	Associated with increased objective response rates and overall survival	Kim Y et al. 2024^120^
Non-small cell lung cancer	Stage IIIA-IV NSCLC patients (*n* = 60)	Metagenomic analysis	Treatment (NCT03013335)	*Akkermansia muciniphila*	Restore the efficacy of PD-1 blockade in an interleukin-12 dependent manner by increasing the recruitment of CCR9^+^CXCR3^+^CD4^+^ T lymphocytes	Vétizou M et al. 2015^121^
Renal cell carcinoma	Patients with stage IV clear cell or non-clear cell histology (*n* = 40)		Treatment (NCT03013335), (NCT01668784), (NCT01375842)			
Urothelial carcinoma	Patients with UC (*n* = 42)		Treatment (NCT01693562)			
Colorectal cancer	AOM/DSS-induced mice; patients with CRC (*n* = 92)	16S rRNA sequencing	Biomarker	*Bacteroides fragilis, Bacteroides thetaiotaomicron*	Anti-cancer effects associated with CTLA-4 blockade therapy	Routy B et al. 2018^122^
Hepatocellular carcinoma	patients (*n* = 4)	Metagenomic analysis	Treatment (NCT04264975)	*Prevotella merdae Immunoactis*	Boosts T cell activity and reduces tumor growth	Derosa L et al. 2022^123^
Esophageal squamous cell carcinoma	patients (*n* = 5)					
Gastric cancer	patients with HCC (*n* = 4)					
Colorectal cancer	AOM/DSS-induced mice; patients with CRC (*n* = 92)	16S rRNA sequencing	Biomarker	Lactobacillus johnsonii	Enhance the efficacy of CD8 + T cell-mediated aPD-1 immunother	Qiu et al. 2022^124^
Lung cancer	NSCLC (*n* = 338) treated with first- or second-line ICIs	Shotgun-metagenomics-based microbiome profiling	Biomarker	*Akkermansia muciniphila*	associated with increased objective response rates and overall survival	Derosa L et al. 2022^123^
Metastatic renal cell carcinoma (mRCC)	Locally advanced or mRCC (*n* = 30) received cabozantinib and nivolumab	Metagenomic sequencing	Treatment (NCT05122546)	*CBM588* (a bifidogenic live bacterial product)	Higher objective response rate (14 of 19, 74% vs. 2 of 10, 20%; *p* = 0.01) and progression-free survival at 6 months in the experimental arms (84% vs. 60%)	Ebrahimi H et al. 2024^125^
Refractory melanoma	Healthy donor FMT with nivolumab or pembrolizumab in patients with advanced melanoma (*n* = 20)	Metagenomics shotgun sequencing and 16S rRNA gene sequencing	Treatment (NCT03772899)	Fecal microbiota transplantation (healthy donor)	The objective response rate was 65% (13 of 20), including four (20%) complete responses	Routy B et al. 2023^126^
AML	Hematopoietic cell transplantation (HCT) recipients (*n* = 74) and AML cohort (*n* = 26) received standardized oral encapsulated FMT	16S rRNA gene sequencing	Treatment (NCT03678493)	Fecal microbiota transplantation	Third-party FMT was safe and ameliorated intestinal dysbiosis, but did not decrease infections	Rashidi A et al. 2023^127^
Colorectal cancer	Patients (*n* = 42) treated with regorafenib plus toripalimab	16S rRNA gene sequencing	Biomarker (NCT03946917)	*Fusobacterium*	Patients with high-abundance Fusobacterium have shorter PFS than those with low abundance (median PFS = 2.0 versus 5.2 months; *p* = 0.002)	Wang et al. 2021^128^
Melanoma	PD-1-refractory melanoma patients (*n* = 16) received anti-PD-1 responder-derived FMT and pembrolizumab	Shotgun metagenomic sequencing	Treatment (NCT03341143	Fecal microbiota transplantation (anti-PD-1 responder)	Reprogrammed the tumor microenvironment to overcome resistance to anti-PD-1	Davar D et al. 2021^129^
Advanced solid malignancies (HNSCC and melonoma)	Patients (*n* = 40) received anti-PD-1 antibodies or anti-PD-1 and anti-CTLA-4 antibodies in combination	16S rRNA gene sequencing and targeted metabolomics	Treatment (NCT03686202)	*Orally delivered 30-species microbial consortium (Microbial Ecosystem Therapeutic 4, MET4)*	Increases in the relative abundance of Enterococcus and Bifidobacterium, associated with ICI responsiveness	Spreafico A et al. 2023^130^
NSCLC or ovarian cancer	Patients (*n* = 38) treated with platinum-based chemotherapy), autologous DC-derived exosomes and CTX	16S rRNA gene sequencing	Biomarker (NCT01159288)	*E. hirae and B. intestinihominis*	Associated with enhanced cognate anticancer immune responses	Daillère R et al. 2016^131^
Refractory advanced cancers	Patients (*n* = 24) received repeat intratumoral injections of SYNB1891 either alone or in combination with atezolizumab	PCR	Treatment (NCT04167137)	*SYNB1891 (Engineered E. coli nissle strain expressing STING agonist)*	Activation of the STING pathway and target engagement	Luke JJ et al. 2023^132^

**Figure 3.**
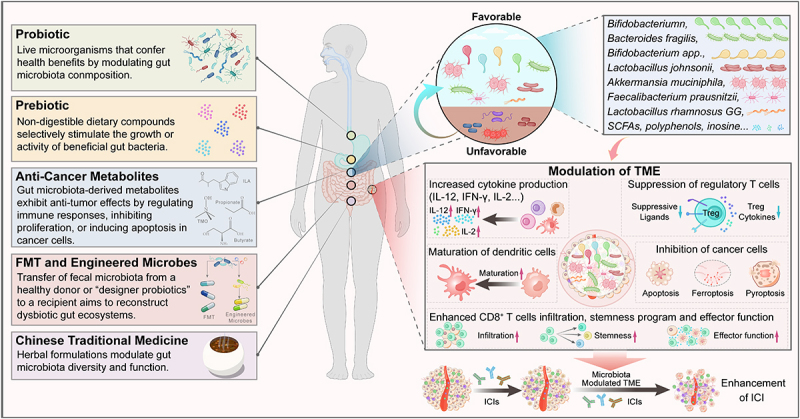
Mechanisms of gut microbiome enhancing effects of cancer therapy. Gut microbiota-driven strategies synergize with cancer therapies by reshaping the TME and augmenting ICB efficacy. Key mechanisms include: (1) Probiotics: Beneficial bacteria (e.g., Akkermansia muciniphila, Bifidobacterium spp., Lactobacillus) enhance dendritic cell (DC) maturation, promote CD8^+^ T-cell infiltration, and boost effector functions via cytokine production (IL-12, IFN-γ). (2) Prebiotics: dietary compounds (e.g., inulin, FOS) increase SCFA production (e.g., butyrate) and inhibit regulatory T cells (Tregs). (3) Anti-cancer metabolites: SCFAs (e.g., butyrate) recruit CCR9^+^CXCR3^+^CD4^+^ T cells and enhance CD8^+^ T-cell stemness. Phenolic acids induce tumor cell apoptosis through mitochondrial dysfunction. (4) FMT and engineered microbes: restores microbial diversity in non-responders, upregulates immunomodulatory metabolites (e.g., punicic acid), and synergizes with ICB (anti-PD-1/CTLA-4) to improve survival. (5) traditional Chinese medicine (TCM): formulations like Wenzi Jiedu recipe remodel gut microbiota, elevate CD8^+^ T-cell proportions, and suppress colorectal cancer (CRC) progression via immune-associated cytokine.

The efficacy of immune checkpoint blockade (ICB) therapies, including anti-PD-1 and anti-CTLA-4 agents, is closely linked to the composition and diversity of the gut microbiome.^124^ Gut microbiome can modulate immune checkpoint inhibitors (ICIs) therapy by enhancing immune cell responses, thus improving cancer treatment efficacy.^133^ Studies have shown that specific bacterial species, such as *Bacteroides fragilis*, enhance the sensitivity to CTLA-4 blockade by promoting Th1 immune responses.^134^ Patients with enriched *Akkermansia muciniphila (A. muciniphila)* exhibit higher response rates to anti-PD-1 therapy, as seen in melanoma and non-small cell lung cancer (NSCLC).^123^ It achieves this by increasing the recruitment of CCR9^+^CXCR3^+^CD4^+^ T lymphocytes into tumor sites, which is crucial for effective immunotherapy.^122^ Additionally, *A. muciniphila* supplementation has been found to restore the efficacy of PD-1 blockade in an interleukin-12-dependent manner.^121^ Emerging research reported that fecal microbiota transplantation (FMT) from responders to non-responders could restore responsiveness to anti-PD-1 therapies, as demonstrated in clinical trials like NCT04264975.^120^ Some specific members of the gut microbiome, such as *Bifidobacterium spp*., have been shown to enhance the efficacy of anti-PD-L1 therapy by promoting antitumor immunity and facilitating T cell infiltration into tumors.^135^ Recent studies elucidate the mechanisms through which the microbiota modulates ICB outcomes.^136^ Supplementation with *Lactobacillus johnsonii* or tryptophan-derived metabolite indole-3-propionic acid (IPA) enhances the efficacy of CD8^+^ T cell-mediated αPD-1 immunotherapy by modulating the stemness program of CD8^+^ T cells and facilitates the generation of progenitor exhausted CD8^+^ T cells (Tpex).^119^ Based on this evidence, distinct bacterial species and genera such as *Faecalibacterium prausnitzii* and *Bifidobacterium* have been found to enhance immunotherapy responses following PD-1/PD-L1 and CTLA-4 blockage, resulting in better clinical outcomes for cancer patients.^137^ Ongoing investigations are exploring how manipulating the gut microbiome can augment ICB efficacy. For example, engineered probiotics producing immune-activating cytokines are being tested in murine tumor models, with promising results in enhancing T-cell infiltration into tumors.^138^ Dietary extracellular vesicles derived from *Lactobacillus rhamnosus* GG (LGG) enhanced anti-PD-1 immunotherapy efficacy against CRC by modulating intestinal immunity and increasing the diversity of beneficial bacteria such as *Lactobacillus*.^139^ These researches demonstrated that the gut microbiome boosts the therapeutic effect of cancer by enhancing immune checkpoint blockade (Figure 3).

### 5.2. Modulation of tumor microenvironment

The gut microbiome influences the TME by modulating immune-related receptors in adaptive immune cells, such as CD80, CD86, and PD-L1, which are crucial for the therapeutic effect of cancer.^123,140^ This interaction between the gut microbiome and adaptive immune cells can lead to immune evasion or suppression, depending on the microbial composition.^141^ Gut bacteria like *Bacteroides fragilis* and *A. muciniphila* enhance dendritic cell maturation and cytokine production (IL-12, IFN-γ), which boost CD8^+^ T-cell activation and tumor cell clearance.^121^ Specific gut bacteria, such as *Bifidobacterium* and *Streptococcus*, have been shown to enhance antitumor immunity by promoting the infiltration and activation of effector CD8^+^ T cells into the TME.^142^ Changes in glycerophospholipid metabolism and the expression of immune-related cytokines such as IFN-γ and IL-2 within the TME have been linked to better treatment outcomes.^143^ These metabolic alterations can enhance systemic and antitumor immunity, thereby improving the effectiveness of PD-1 antibody therapy.^144^ Some specific microbial metabolites suppress regulatory T cells (Tregs), mitigating their immunosuppressive effects in the TME,^145^ and the other specific microbial metabolic byproducts can significantly influence the efficacy of cancer immunotherapies through enhancement of anti-tumor immunity.^89^ For instance, certain gut bacteria produce metabolites like inosine, which can enhance T-cell differentiation and effector functions through pathways such as the inosine-A2AR-cAMP-PKA pathway, thereby supporting anti-tumor immunity.^146^ This indicates that the gut microbiome and its metabolites can enhance anti-tumor immunity and improve tumor treatment efficacy by modulating the quantities of various adaptive immune cells, as well as the expression of cytokines in the TME (Figure 3).

### 5.3. Probiotic and prebiotic applications

Probiotics and prebiotics are being increasingly recognized as adjunctive therapies in oncology.^147^ Dietary interventions and probiotics have been shown to effectively modulate the gut microbiome, which in turn can enhance cancer treatment outcomes in esophageal cancer.^148^ Probiotics like *Lactobacillus rhamnosus* and *Bifidobacterium longum* can reduce inflammation, promote gut barrier integrity, enhance mucosal immunity and stimulate the production of anti-inflammatory cytokines (IL-10) while suppressing pro-inflammatory cytokines (IL-6, TNF-α).^149^ Strains like *Lactobacillus casei* and *Bifidobacterium breve* mitigate side effects like mucositis and diarrhea in patients undergoing chemoradiotherapy through reducing inflammation, inducing apoptosis in tumor cells, inhibiting metastasis, and promoting epithelial regeneration.^150,151^ Studies have demonstrated that the diminished effectiveness of immunotherapy in cancer patients is associated with a reduction in *A. muciniphila* levels and supplementation with SCFA-producing probiotics exerts tumor-suppressive effects with potential in enhancing the efficacy of radiation therapy and immunotherapy in both preclinical and clinical settings.^152,153^ Recent studies have developed some engineered probiotics, such as targeting the Adenosine-Mediated Metabolic Immune Checkpoint, providing not only a new idea for building a safe and efficient live bacteria delivery platform with dual immunomodulatory abilities but also a novel approach for enhancing cancer immunotherapy.^154–156^ Prebiotics, such as inulin and fructooligosaccharides (FOS), selectively stimulate the growth of beneficial bacteria.^157^ They increase SCFA production, particularly butyrate, which has been shown to suppress histone deacetylase (HDAC) activity, leading to reactivation of tumor suppressor genes.^158^ In addition, synbiotics, which are combinations of prebiotics and probiotics, have been studied for their effects during neoadjuvant chemotherapy in esophageal cancer patients.^159^ A randomized study highlighted the potential of synbiotics to reduce adverse events associated with chemotherapy.^147^ Translational research, such as Clinical trials (NCT04843504), is evaluating the combined use of probiotics and prebiotics in enhancing chemotherapy tolerance, reducing adverse effects, and improving immune checkpoint inhibitor efficacy.^160^ Therefore, the applications of probiotics and prebiotics will become an important strategy for assisting cancer therapy (Figure 3).

### 5.4. Anti-cancer metabolites

Gut microbiota-derived metabolites, such as SCFAs, polyphenols, and tryptophan derivatives, are emerging as therapeutic agents.^161^ For instance, SCFAs produced by gut bacteria can stimulate chemokine production by CRC cells, thereby favoring the recruitment of beneficial T cells into tumor tissues.^162^ Meanwhile, phenolic acids, produced via microbial fermentation, induce mitochondrial dysfunction in tumor cells, leading to apoptosis.^163^ Metabolites produced by specific gut bacteria are expected to translate to therapeutic agents of cancer (Figure 3).

### 5.5. Traditional Chinese medicine (TCM)

Recently, many studies have shown that traditional Chinese medicine can safely and effectively improve the efficacy of antitumor drugs, potentially through remodeling the TME by regulating gut microbiome.^164^ Certain TCM formulations, such as Wenzi Jiedu Recipe (WJR), have been found to inhibit CRC progression by remodeling the gut microbiome and enhancing anti-tumor immunity through increased proportions of CD8^+^ T cells and expression of immune-associated cytokines.^124^ Therefore, it is an effective strategy to explore new immunotherapies for cancer to clarify the immunoregulatory effects of TCMs on intestinal microbiota (Figure 3).

### 5.6. Fecal microbiota transplantation

Microbiota-targeted interventions are at the forefront of oncology research, including Fecal Microbiota Transplantation (FMT)^126^ (Figure 3). Clinical trials (e.g., NCT04130763) show that FMT enhances chemotherapy efficacy in CRC by restoring microbiota diversity and reducing gastrointestinal toxicity induced by 5-Fluorouracil/Oxaliplatin.^165^ Studies have demonstrated that combining FMT with anti-PD-1 therapy leads to superior survival rates and tumor control compared to either treatment alone.^166^ Metabolomic analysis revealed that FMT upregulates metabolites such as punicic acid and aspirin, which may promote the response to anti-PD-1 therapy through their immunomodulatory functions.^167^ FMT is also being tested as an adjunct to immune checkpoint inhibitors, with preliminary results showing improved patient response rates in NSCLC.^168^ Engineered microbes, like synthetic biology, enables the creation of “designer probiotics” that produce anti-cancer agents directly within the TME.^169^ These engineered microbes can secrete cytokines or cytotoxic molecules by selectively targeting tumor cells.^170^ Combining gut microbiome modulation with other therapies, such as chemotherapy or ginseng polysaccharides, has shown promising results in sensitizing patients to anti-PD-1/PD-L1 immunotherapy. For example, ginseng polysaccharides have been found to increase microbial metabolites that suppress regulatory T cells and induce effector T cells, thereby enhancing the antitumor response.^171^ Although FMT has been a widely used approach in cancer treatment, but its long-term stability is challenged due to the influence of the intestinal microenvironment.

## 6. Challenges and future research directions

### 6.1. Overcoming methodological limitations

Despite rapid advancements in microbiome research, methodological inconsistencies pose significant barriers to progress. The first challenge is the sampling variability. The microbial composition in feces can only reflect the general trend of gut microbiome composition. In fact, the precise distribution of gut microbiome in different parts of the digestive tract varies, and their roles and functions in maintaining intestinal homeostasis are also different. Therefore, the representativeness of fecal specimens in reflecting the composition of gut microbiome faces challenges. In addition, differences in stool sample collection, preservation, and sequencing methods lead to inconsistent results. For instance, studies comparing CRC-associated dysbiosis often report divergent microbial profiles due to protocol variations.^172^ Another methodological limitation is standardized bioinformatics. The lack of standardized pipelines for analyzing metagenomic data limits cross-study comparability. Integrating AI-based tools to harmonize data processing could mitigate these discrepancies.

### 6.2. Expanding multi-omics integration

Multi-omics approaches, combining metagenomics, metabolomics, and transcriptomics, offer a holistic view of microbial-host interactions. For instance, profiling microbial metabolites by Metabolomics, such as SCFAs and bile acids, provides insights into their roles in tumor biology. Integration with the advanced techniques like mass spectrometry enables precise quantification of these metabolites.^64^ In addition, integration of host transcriptomic data with microbial profiles can reveal novel pathways by which dysbiosis impacts tumor progression.^173^

### 6.3. Ethical and regulatory challenges

The clinical application of microbiota-based therapies, such as FMT, raises ethical and regulatory concerns. The donor selection is very important for ensuring donor microbiota safety. Therefore, cases of pathogen transmission underscore the need for stringent donor screening protocols.^174^ In addition, the long-term effectiveness and safety of FMT are also issues of concern. The impact of introducing foreign microbial communities on host health and its effectiveness remains poorly understood, necessitating long-term monitoring in clinical trials.

### 6.4. Future research directions

Although significant progress has been achieved in the study of gut microbiome, current research is still in its early stages. Especially regarding the roles of gut microbiome in the prevention and treatment of cancers, there is still a long way to go. The key areas warranting further investigation include:

1. Causality vs. Correlation: Currently, most researches of the roles of gut microbiome in cancer suppression are cross-sectional or correlative study. In future, employing germ-free animal models and longitudinal human studies will help establish causal relationships between the interventions and outcomes.
2. Synthetic Microbiota: The survival of gut microbiome depends on the gut microenvironment. If it is difficult to continuously change the composition of the gut microbiome, then it is feasible to alter the function and metabolism of specific gut bacteria using genetic engineering. Therefore, engineering microbial consortia with precise functions, such as producing anti-tumor metabolites, holds promise for personalized tumor treatment.
3. Gut microbiome and Radiotherapy: Although FMT has been applied to reduce radiotherapy-associated toxicity in colorectal cancer treatment,^136^ the roles and mechanisms of gut microbiome in radiotherapy of cancer remain unclear. The next step is exploring how microbiota influence radiotherapy responses could lead to novel strategies to mitigate radiation-induced toxicities.
4. Interaction between gut microbiota: In order to maintain the balance of gut microbiota, there are complex interactions among various bacterial species. Exploring the interactions between gut bacteria in various disease will provide new strategies for regulating the gut microenvironment.
5. Biological rhythms and gut microbiome: In recent years, there have been an increasing number of studies on the biological rhythm-dependent mutual influence of microbiome and host’s organism.^175^ The abnormalities in the microbiome as well as any disturbances in the host’s circadian rhythms are associated with numerous diseases including cancers.^176–178^ Since abnormal circadian rhythms can accelerate the occurrence and development of cancer by reshaping the gut microbiome, it is feasible to inhibit the initiation and development of cancers by correcting biological rhythms and improving the dysfunction of gut microbiome in the future.
6. Gut microbiome-related animal model: The development of animal models for various human diseases is dependent on the involvement of gut microbiome. Establishing animal models related to gut microbiota that can be stably inherited is also an important research direction in the future.
7. Relationship between the localization of gut microbiome and cancers: The ileum and cecum exhibit distinct microbiome–tumor relationships due to differences in microbial ecology, host physiology, and immune activity. By analyzing the differences in microbial communities in different regions of CRC (tumor core, margin, normal mucosa) through spatial metagenomics, it was found that the position of the intestinal segment significantly affects the composition of the microbial community.^179^ In the distal colon, it has been shown that *Bacteroidetes* are enriched in the lumen, while *Firmicutes* are enriched in the mucus layer and crypts.^180^ In the mouse cecum, *Bacteroidetes* is increased in relative abundance from the cecal tip to the base.^180^ The gut immune system varies in function and composition across different regions. This spatial restriction allows it to adapt to specific microbiota and environmental factors in each area.^181^ Future research should focus on region-specific microbial biomarkers and therapies for cancers.

## 7. Conclusion

The pathogenesis of cancers includes a combination of genetic factors and environmental exposures, as well as the disruption of homeostasis, and the gut microbiome plays crucial roles in maintaining the homeostasis of host. In terms of time dimension, the gut microbiome remains in a dynamic equilibrium throughout the course of life. This dynamic balance of gut microbiome can be disrupted at any time, sometimes it promotes the occurrence and development of cancer, and sometimes it inhibits the occurrence of cancer, depending on the interaction between the gut microbiome and host homeostasis. Imbalance of gut microbiome has a double-edged sword effect on cancers. Under the same conditions, some intestinal bacteria promote the occurrence and development of cancers, while the others may play a completely opposite role. It is worth noting that one or multiple strains of gut microbiota may promote the occurrence of one disease while inhibiting the occurrence of another disease. For example, we have reported that an enriched specific genera, named *Prevotella*, can simultaneously promote AD onset and inhibit CRC tumorigenesis. It is indicated that there is a complex regulatory network between the gut microbiota and its host. Gut microbiome exerts profound influence on suppression of cancers via inhibiting the initiation of cancer, delaying the cancer progression and boosting the therapeutic effect of cancer, and the complex mechanisms involve inflammation, metabolism, and immune modulation. Leveraging these insights, microbiota-targeted therapies offer transformative potential in oncology, from prevention to personalized treatment strategies. However, addressing methodological and ethical challenges is imperative for translating microbiota research into clinical practice. The integration of advanced multi-omics approaches and synthetic biology promises a new frontier in cancer therapeutics. The key areas warranting further investigation also include the interaction between gut microbiome, synthetic microbiota, development of gut microbiota-related animal model, and the roles of gut microbiome in radiotherapy and biological rhythms.

## Data Availability

All data are included in the manuscript.
